# Beyond Cartesian Dualism: Neuropsychiatric Presentation of Fahr’s Syndrome Secondary to Pseudohypoparathyroidism: A Case Report

**DOI:** 10.1192/j.eurpsy.2025.524

**Published:** 2025-08-26

**Authors:** M. Fatima, A. Aslam, F. A. Randhawa, Z. Khalid, S. Mumtaz, M. S. Alam, A. Nawaz

**Affiliations:** 1 Psychiatry, Dublin North Mental Health Service, Dublin, Ireland; 2 Neurology; 3 Endocrinology/ General Medicine; 4 King Edward Medical University , Lahore, Pakistan

## Abstract

**Introduction:**

Fahr’s Syndrome is a rare disorder characterized by abnormal brain calcium deposits presenting as extrapyramidal signs, cognitive impairment, movement disorders, and psychiatric symptoms. Though well documented in association with hypoparathyroidism, its presentation secondary to pseudohypoparathyroidism (PHP) is exceptionally unusual.

**Objectives:**

To report a rare neuropsychiatric presentation of Fahr’s Syndrome secondary to PHP.

**Methods:**

We report this rare case following the CARE Case Report guidelines.

A 25-year-old female was admitted to the neurology ward after a referral from the Psychiatry clinic for dysarthria, involuntary movements affecting gait and daily activities, worsening psychomotor slowing for five years & depressive symptoms for around a year. She had difficulty controlling anger for 2.5 years & 2 impulsive attempts at self-harm in the past 6 months with no suicidal intent in which she tried to slash her wrist & jump off the rooftop over verbal confrontations with family (Image1).

**Results:**

Neuroimaging revealed bilateral, symmetrical calcifications in basal ganglia, thalami, red nuclei, the gray matter of bilateral occipital and frontal lobes, and cerebellar hemispheres (Image 2). Image 3 shows MRI Brain (with contrast) axial section images: Altered hyperintense signals in bilateral basal ganglia & red nuclei of midbrain on T1WI, hypointense signals on T2/FLAIR, and Blooming artifacts on SWAN images.

She was diagnosed with PHP based on hypocalcemia, hyperphosphatemia, high intact parathormone (PTH), and normal Serum Vitamin D levels. The absence of Albright’s Hereditary Osteodystrophy (AHO) phenotype and normal thyroid profile suggested PHP-type 2/1B but we couldn’t perform the Ellsworth Howard test to distinguish due to the unavailability of synthetic PTH. She was treated with oral calcium supplements, oral active vitamin D (CALCITRIOL or 1, 25-DIHYDROXYCHOLECALCIFEROL), CALCIUM ACETATE, AMANTADINE, ALFACALCIDOL, and ESCITALOPRAM leading to significant improvement in depressive symptoms, and normocalcemia at one-year follow-up.

**Image 1:**

**Figure:**
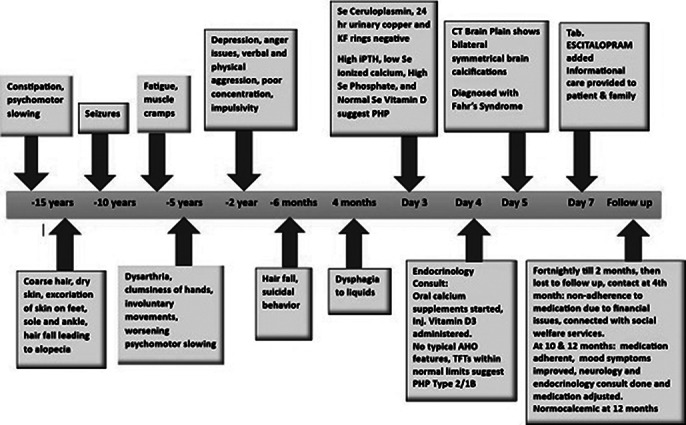

**Image 2:**

**Figure:**
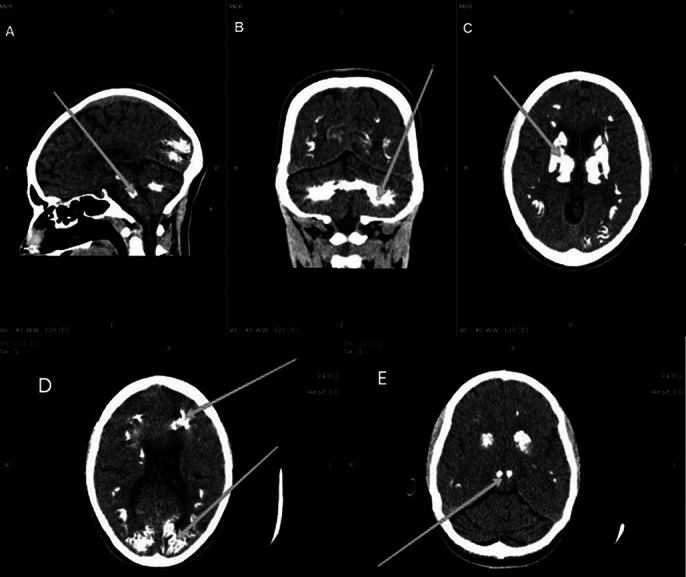

**Image 3:**

**Figure:**
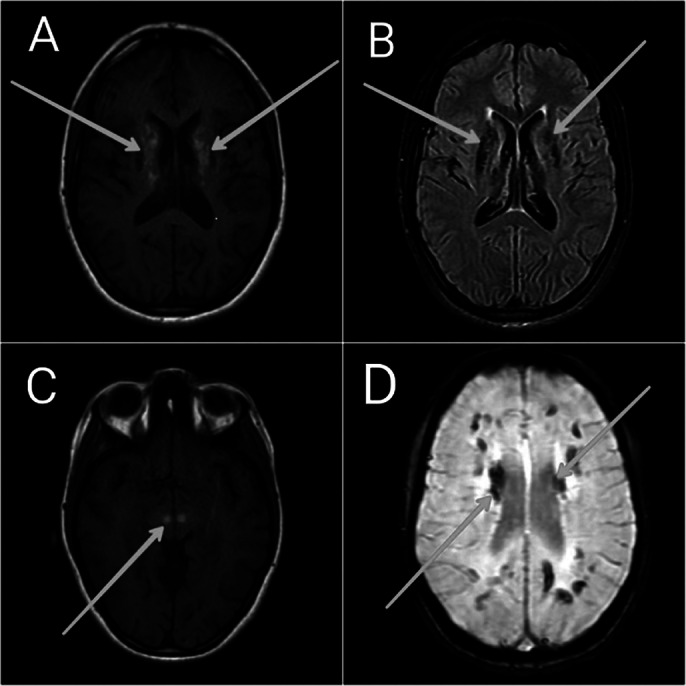

**Conclusions:**

The diagnosis and management of rare neuropsychiatric syndromes maybe challenging in low-resource settings and timely diagnosis and management necessitate looking beyond the mind-body dualism and ensuring a multidisciplinary approach. Our patient’s neurological features were overlooked at a specialist mental healthcare facility for 5 years which accentuates the significance of thorough neurological assessment during psychiatric evaluations and of incorporating neuropsychiatry and psycho-neuro-endocrinology into the training and practice of psychiatry and neurology in Pakistan. This case also provides insights into the economic barriers to the management of neuropsychiatric disorders in a lower middle-income country (LMIC), where high out-of-pocket expenditure on treatment may result in loss of follow-up.

**Disclosure of Interest:**

None Declared

